# Development and validation of nomograms to predict clinical outcomes of preeclampsia

**DOI:** 10.3389/fendo.2024.1292458

**Published:** 2024-03-14

**Authors:** Yan Xia, Yao Wang, Shijin Yuan, Jiaming Hu, Lu Zhang, Jiamin Xie, Yang Zhao, Jiahui Hao, Yanwei Ren, Shengjun Wu

**Affiliations:** ^1^ Department of Clinical Laboratory, Sir Run Run Shaw Hospital, Zhejiang University School of Medicine, Hangzhou, China; ^2^ Key Laboratory of Precision Medicine in Diagnosis and Monitoring Research of Zhejiang Province, Hangzhou, China; ^3^ Department of Medical Oncology, Sir Run Run Shaw Hospital, Zhejiang University School of Medicine, Hangzhou, China; ^4^ School of Medical Technology and Information Engineering, Zhejiang Chinese Medical University, Hangzhou, China; ^5^ Department of Gynaecology and Obstetrics, Sir Run Run Shaw Hospital, Zhejiang University School of Medicine, Hangzhou, China

**Keywords:** pre-eclampsia, nomogram, biomarker, predictive model, peripheral biomarkers

## Abstract

**Background:**

Preeclampsia (PE) is one of the most severe pregnancy-related diseases; however, there is still a lack of reliable biomarkers. In this study, we aimed to develop models for predicting early-onset PE, severe PE, and the gestation duration of patients with PE.

**Methods:**

Eligible patients with PE were enrolled and divided into a training (*n* = 253) and a validation (*n* = 108) cohort. Multivariate logistic and Cox models were used to identify factors associated with early-onset PE, severe PE, and the gestation duration of patients with PE. Based on significant factors, nomograms were developed and evaluated using the area under the curve (AUC) and a calibration curve.

**Results:**

In the training cohort, multiple gravidity experience (*p* = 0.005), lower albumin (ALB; *p* < 0.001), and higher lactate dehydrogenase (LDH; *p* < 0.001) were significantly associated with early-onset PE. Abortion history (*p* = 0.017), prolonged thrombin time (TT; *p* < 0.001), and higher aspartate aminotransferase (*p* = 0.002) and LDH (*p* = 0.003) were significantly associated with severe PE. Abortion history (*p* < 0.001), gemellary pregnancy (*p* < 0.001), prolonged TT (*p* < 0.001), higher mean platelet volume (*p* = 0.014) and LDH (*p* < 0.001), and lower ALB (*p* < 0.001) were significantly associated with shorter gestation duration. Three nomograms were developed and validated to predict the probability of early-onset PE, severe PE, and delivery time for each patient with PE. The AUC showed good predictive performance, and the calibration curve and decision curve analysis demonstrated clinical practicability.

**Conclusion:**

Based on the clinical features and peripheral blood laboratory indicators, we identified significant factors and developed models to predict early-onset PE, severe PE, and the gestation duration of pregnant women with PE, which could help clinicians assess the clinical outcomes early and design appropriate strategies for patients.

## 1 Introduction

Preeclampsia (PE), which typically occurs after 20 weeks of gestation, is one of the most severe pregnancy-related diseases. It is characterized by sudden-onset hypertension and is accompanied by at least one of the following complications: proteinuria and maternal organ dysfunction (1). Globally, there are an estimated 4 million women newly diagnosed with PE each year, resulting in the death of more than 70,000 women and 500,000 newborns, making it the leading cause of maternal and perinatal morbidity and mortality (2, 3).

The heterogeneity of PE as clinical presentation and outcome varies between different subtypes. Patients with early-onset PE (<34 weeks of gestation) always present more severe clinical complications and an enrichment of metabolism-related pathways in the transcriptional profile compared to those with late-onset PE (≥34 weeks of gestation) (4, 5). Patients with PE are also at risk of rapid deterioration and severe disease, including eclampsia, stroke, HELLP (hemolysis, elevated liver enzymes, and low platelets) syndrome, placental abruption, renal function failure, and pulmonary edema, without receiving timely treatment (6). The management of PE consists of monitoring perinatal blood pressure and controlling complications through pharmacological intervention. Currently, timely delivery of the fetus is the only definitive treatment for PE; however, it may cause the babies of women with early-onset PE or severe symptoms to have increased risks of preterm birth, perinatal death, neurodevelopmental delay, and later cardiovascular and metabolic diseases (2). Therefore, early identification of the occurrence of PE, especially early-onset PE and severe PE, and prediction of gestation duration are of utmost importance to minimize adverse perinatal events both in pregnant women and in fetuses.

Three checklists from the International Society for the Study of Hypertension in Pregnancy (ISSHP) (3), the American College of Obstetricians and Gynecologists (ACOG) (7), and the National Institute for Health and Care Excellence (NICE) (8) are broadly used in clinical practice to assess the risk of PE occurrence; however, all risk factors derived from clinical features and their predictive power for PE are weak (9). Recently, increased numbers of biomarkers from peripheral blood have been identified to predict pregnant women with a high risk of PE at an early stage. Soluble fms-like tyrosine kinase 1 (sFlt-1) and placental growth factor (PlGF) are a pair of anti- and pro-angiogenic factors (respectively) found significantly unbalanced in PE (10). The PROGNOSIS trial demonstrated that, in women with a sFlt-1/PlGF ratio lower than 38, the likelihood of developing PE over the next week could accurately be ruled out, with a 99.3% negative predictive value (11). In addition, a series of novel placental- and endothelial-derived nucleic acid (mainly RNA) and proteins were also discovered for PE, including extravillous trophoblast signature (*MMP11*, *SLC6A2*, and *IL18BP*) (12), the chromosome 19 miRNA cluster (combination of miR-517-5p, miR520a-5p, and miR-525-5p) (13, 14), placental protein 13 (PP13) (15), pregnancy-associated plasma protein A (PAPP-A) (16), and vascular cell adhesion molecule-1 (VCAM-1) (17). However, the efficacy of a single biomarker from peripheral blood in the accurate diagnosis of PE is inadequate, and the majority of studies lacked validation. Hence, the development and validation of a predictive model based on multiple indicators consisting of clinical characteristics and laboratory parameters could be helpful in clinical practice. In the recent decade, several predictive models have been developed based on a series of risk factors. For example, by combining gestational age, chest pain or dyspnea, oxygen saturation, platelet count, and the creatinine and aspartate transaminase concentrations, the fullPIERS model could identify the risk of fatal or life-threatening complications in women with PE within 48 h of hospital admission (18). In addition, another study constructed a machine learning model for the prediction of PE in the first trimester based on the mean arterial blood pressure, uterine artery pulsatility index, PlGF, and PAPP-A (19). However, these models could not provide an exact probability for PE occurrence and the delivery of pregnant women at a certain time. A nomogram is a predictive tool to evaluate the clinical outcomes of patients by quantifying the probability based on easily accessed variables, which is widely used in patients with cancer and other chronic diseases, even in patients with coronavirus disease 2019 (20–22).

In this study, based on the clinical characteristics and peripheral blood laboratory indicators of patients with PE, we aimed to identify biomarkers for the early diagnosis of PE with early-onset and severe symptoms, predict the gestation duration, and construct a model for each clinical outcome, which could help clinicians recognize and manage patients with PE in the early stage of the disease and improve the clinical prognosis for pregnant women and infants.

## 2 Methods

### 2.1 Study population

This study retrospectively enrolled patients from Sir Run Run Shaw Hospital, Zhejiang University School of Medicine, China, in January 2017 and December 2022. Eligible populations were diagnosed with PE according to the “Diagnosis and treatment of hypertension and preeclampsia in pregnancy: a clinical practice guideline in China (2020)” (23). The detailed diagnostic criteria for PE were as follows: pregnant women with a systolic blood pressure higher than 140 mmHg and/or a diastolic blood pressure higher than 90 mmHg after 20 weeks of gestation, accompanied by any of the following symptoms: 1) urine protein ≥0.3 g/24 h or a urine protein/creatinine ratio ≥0.3 and 2) any dysfunction of important organs such as the heart, lung, liver, kidney, blood system, digestive system, and nervous system or involvement of placenta–fetus. The exclusion criteria were as follows: 1) patients with preexisting hypertension, immune disorders, and maternal organ dysfunction (such as hematopoietic, hepatic, and renal dysfunction) and 2) patients without complete maternal or infant records.

A total of 361 eligible patients were enrolled. This study was approved by the Ethics Committee of Sir Run Run Shaw Hospital (approval no. 2023-0248).

### 2.2 Data collection

Data on the clinical characteristics and laboratory indicators were collected from the electronic medical record of each patient. Clinical characteristics included age, history of parity, gravidity and abortion, multiple pregnancy, and regularity of the menstrual cycle. Laboratory indicators were collected at 20 weeks of gestation, which included white blood cell (WBC) count, red blood cell (RBC) count, hemoglobin (Hb), hematocrit, mean corpuscular volume (MCV), red blood cell distribution width (RDW), absolute neutrophil count (ANC), absolute lymphocyte count (ALC), absolute monocyte count (AMC), absolute eosinophil count (AEC), absolute basophil count (ABC), platelet count (PC), platelet distribution width (PDW), mean platelet volume (MPV), thrombocytocrit, thrombin time (TT), prothrombin time (PT), activated partial thromboplastin time (APTT), international normalized ratio (INR), fibrinogen, alanine aminotransferase (ALT), aspartate aminotransferase (AST), alkaline phosphatase (ALP), albumin (ALB), lactate dehydrogenase (LDH), serum amyloid A (SAA), total bile acid (TBA), and C-reactive protein (CRP).

### 2.3 Clinical outcomes

According to the gestational age at PE diagnosis, the patients were classified into an early-onset PE (<34 weeks) and a late-onset PE (≥34 weeks) group (24). The diagnostic criteria for severe PE were as follows: 1) continuously increasing blood pressure (systolic pressure ≥160 mmHg and/or diastolic pressure ≥110 mmHg); 2) persistent headache, visual disturbance, or other central nervous system abnormalities; 3) persistent upper abdominal pain, subcapsular hematoma, or liver rupture; 4) abnormal elevation of the AST and ALT levels; 5) impaired renal function—urinary protein quantification ≥2 g/24 h, oliguria, or serum creatinine level >106 μmol/L; 6) hypoproteinemia with ascites, pleural effusion, or pericardial effusion; 7) PC decreasing continuously and lower than 100 × 10^9^/L, microvascular hemolysis, anemia, elevated LDH level, or jaundice; 8) heart failure; 9) pulmonary edema; and 10) fetal growth restriction, oligohydramnios, intrauterine fetal death, and placental abruption. A PE patient with one of the above symptoms was diagnosed as severe PE (23). The gestation duration was defined as the period between the last menstrual period and delivery.

### 2.4 Statistical analysis

Normality of the continuous variables was assessed using the Shapiro–Wilk test. Normally distributed variables were expressed as the mean ± standard deviation (SD), with significance analyzed using Student’s *t*-test. Non-normally distributed variables were expressed as the median and interquartile range (IQR), with significance analyzed using the Mann–Whitney *U* test. Categorical variables were expressed as frequency and percentage, with significance analyzed using the chi-square test.

The whole study population was randomly divided into a training cohort and a validation cohort at a 7:3 ratio using a random sampling method (“Caret” R package). In the training cohort, univariate and multivariate logistic regression models (forward) were used to identify significant variables related to early-onset PE and severe PE (*p* < 0.05), and odds ratios (ORs) and 95% confidence intervals (CIs) were calculated (“glmnet” R package). Univariate and multivariate Cox proportional hazard regression models (forward) were used to identify significant variables related to gestation duration, and hazard ratios (HRs) and 95% CIs were calculated (“survival” R package). The results of the multivariate model were visualized using the “forestplot” R package. The curve of gestation duration was constructed using the Kaplan–Meier method and the log-rank test.

Based on the significant variables in the multivariate model, three nomograms were developed to predict the probability of early-onset PE, severe PE, and delivery at 26, 28, 30, 32, 34, 36, and 38 weeks for each patient with PE (“rms” R package). The predictive performance and the discriminative ability of each nomogram were assessed using 1,000 bootstrap resamples to obtain the concordance index (C-index) and the area under the receiver operating characteristic (ROC) curve (AUC). Calibration curves were used to evaluate the consistency between the predicted probabilities of the nomograms and the actual clinical outcomes. Decision curve analysis (DCA) was performed to evaluate the clinical utility of the nomograms. The above methods were then applied to validate the performance of the nomograms in the validation cohort.

All statistical analyses were performed using R software version 3.6.0. All tests were two-sided, and a *p* < 0.05 was considered statistically significant.

## 3 Results

### 3.1 Patient characteristics

**Figure 1:**
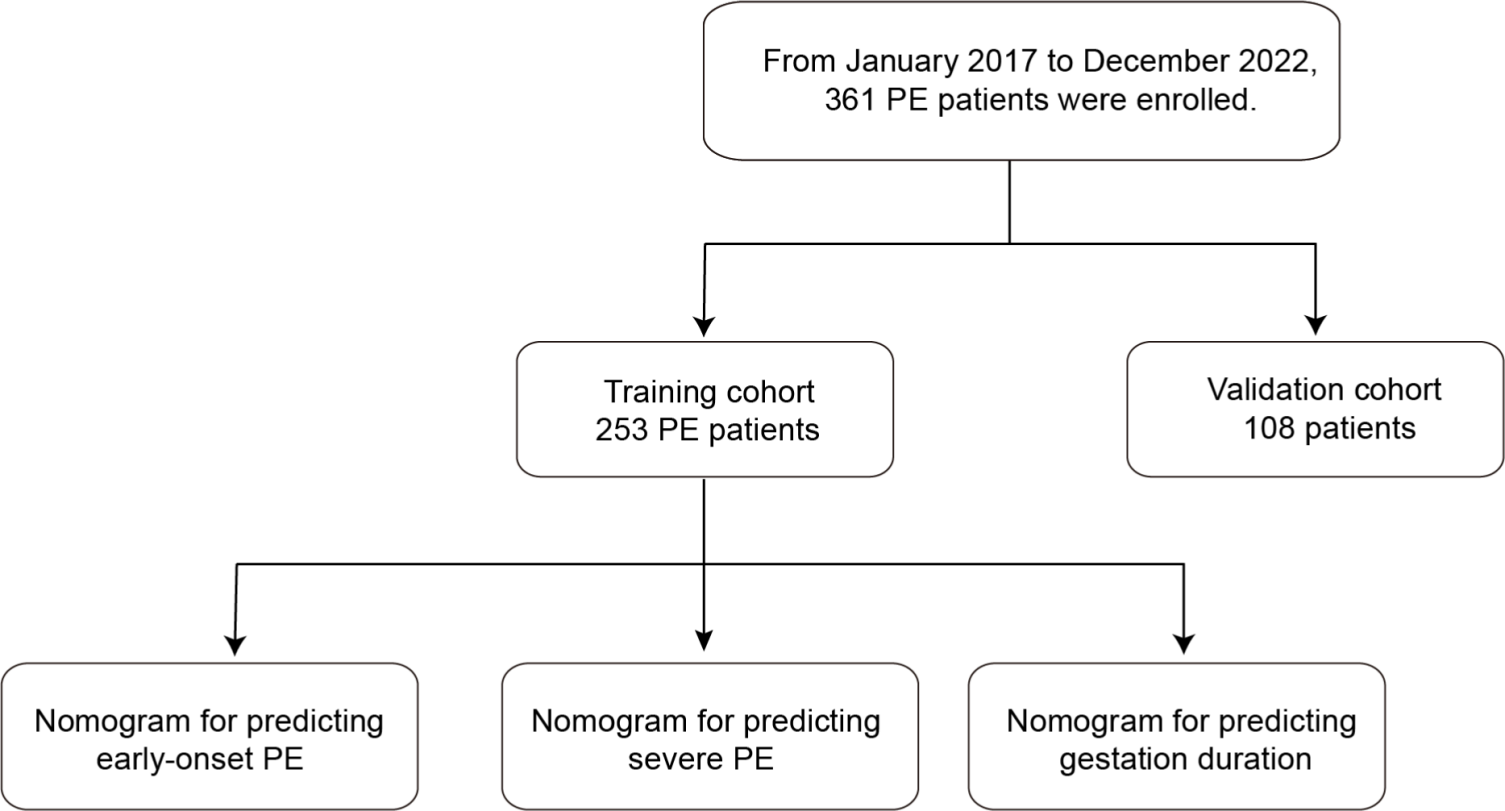
Workflow of the study.

The workflow is shown in **Figure 1**. After random sampling, a total of 361 patients with PE were allocated to the training cohort (*n* = 253) and the validation cohort (*n* = 108). The clinical characteristics and laboratory indicators between the two cohorts were basically balanced (**Table 1**). In the training cohort, the median age was 32.0 years (range, 29.0–35.0 years). A total of 135 patients (53.4%) had multiple gravidity experience, while 188 patients (74.3%) were primiparas. There were 198 patients (78.3%) who had a single pregnancy, 116 patients (45.8%) had a history of abortion, and 221 patients (87.4%) had irregular menstruation.

**Table 1:** Baseline clinical characteristics and laboratory parameters.

Variable	Total patients (n=361)	Training cohort (n=253)	Validation cohort (n=108)	P-value
**Clinical characteristics**				
Age, years (IQR)	32.0 (29.0, 36.0)	32.0 (29.0, 35.0)	32.0 (28.0, 37.0)	0.641
Gravidity				1.000
1	168 (46.5%)	118 (46.6%)	50 (46.3%)	
≥2	193 (53.5%)	135 (53.4%)	58 (53.7%)	
Parity				0.176
Primipara	260 (72.0%)	188 (74.3%)	72 (66.7%)	
Multipara	101 (28.0%)	65 (25.7%)	36 (33.3%)	
Abortion				0.896
No	197 (54.6%)	137 (54.2%)	60 (55.6%)	
Yes	164 (45.4%)	116 (45.8%)	48 (44.4%)	
Gemellary pregnancy				1.000
No	283 (78.4%)	198 (78.3%)	85 (78.7%)	
Yes	78 (21.6%)	55 (21.7%)	23 (21.3%)	
Menstrual regularity				0.398
No	50 (13.9%)	32 (12.6%)	18 (16.7%)	
Yes	311 (86.1%)	221 (87.4%)	90 (83.3%)	
Early-onset PE				0.716
No	278 (77.0%)	193 (76.3%)	85 (78.7%)	
Yes	83 (23.0%)	60 (23.7%)	23 (21.3%)	
Severe PE				0.455
No	178 (49.3%)	121 (47.8%)	57 (52.8%)	
Yes	183 (50.7%)	132 (52.2%)	51 (47.2%)	
**Laboratory parameters**				
WBC, ×10⁹/L	9.70 (8.50, 11.5)	9.80 (8.50, 11.5)	9.65 (8.65, 11.1)	0.958
RBC, ×10^12^/L	3.82 (3.55, 4.09)	3.81 (3.58, 4.08)	3.83 (3.55, 4.12)	0.927
Hb, g/L	108 (12.0, 121)	108 (11.9, 121)	108 (12.0, 121)	0.921
Hematocrit, %	35.1 (33.0, 36.9)	35.2 (33.0, 36.9)	34.5 (33.0, 36.7)	0.454
MCV, fL	92.0 (88.7, 94.8)	91.7 (88.7, 94.6)	92.5 (88.7, 95.0)	0.652
PC, ×10⁹/L	215 (178, 256)	212 (176, 253)	228 (185, 261)	0.187
ANC, ×10⁹/L	7.33 (6.20, 8.84)	7.33 (6.20, 9.00)	7.30 (6.18, 8.52)	0.834
ALC, ×10⁹/L	1.70 (1.45, 2.00)	1.70 (1.40, 2.00)	1.71 (1.50, 2.00)	0.343
AMC, ×10⁹/L	0.51 (0.42, 0.65)	0.50 (0.40, 0.63)	0.52 (0.45, 0.66)	0.310
AEC, ×10⁹/L	0.09 (0.04, 0.10)	0.08 (0.04, 0.10)	0.10 (0.06, 0.11)	0.234
ABC, ×10⁹/L	0.02 (0.00, 0.03)	0.02 (0.00, 0.03)	0.02 (0.00, 0.03)	0.842
RDW, %	13.4 (13.0, 14.0)	13.5 (13.0, 14.0)	13.3 (12.9, 13.8)	0.302
PDW, %	16.7 (16.2, 17.3)	16.8 (16.3, 17.3)	16.6 (16.0, 17.2)	0.562
MPV, fL	8.50 (7.80, 9.30)	8.50 (7.80, 9.30)	8.45 (7.77, 9.15)	0.615
Thrombocytocrit, %	0.18 (0.16, 0.22)	0.18 (0.16, 0.21)	0.20 (0.16, 0.22)	0.203
PT, s	12.4 (12.1, 12.8)	12.4 (12.1, 12.8)	12.4 (12.1, 12.9)	0.476
INR	0.93 (0.90, 0.97)	0.93 (0.91, 0.96)	0.94 (0.90, 0.98)	0.428
APTT, s	31.5 (29.9, 33.0)	31.6 (29.9, 33.1)	31.4 (30.1, 32.8)	0.991
TT, s	15.4 (14.8, 16.1)	15.4 (14.8, 16.1)	15.4 (14.8, 15.9)	0.791
Fibrinogen, g/L	4.69 (0.77)	4.69 (0.76)	4.68 (0.79)	0.895
ALT, U/L	13.0 (10.0, 20.0)	13.0 (10.0, 20.0)	12.0 (9.00, 20.2)	0.345
AST, U/L	18.0 (15.0, 24.0)	18.0 (15.0, 25.0)	17.0 (15.0, 21.0)	0.608
ALP, U/L	72.0 (59.0, 89.0)	72.0 (61.0, 91.0)	73.0 (57.0, 87.0)	0.427
Albumin, g/L	34.6 (32.9, 36.5)	34.9 (32.9, 37.0)	34.2 (33.0, 35.7)	0.058
LDH, U/L	152 (137, 175)	154 (138, 178)	148 (134, 165)	0.115
SAA, mg/L	4.80 (3.10, 7.80)	4.80 (3.20, 7.80)	5.15 (3.00, 7.78)	0.852
TBA, μmol/L	2.48 (1.61, 3.68)	2.50 (1.51, 3.68)	2.40 (1.79, 3.65)	0.436
CRP, mg/L	3.00 (1.80, 5.60)	3.00 (1.80, 5.50)	3.10 (1.78, 5.95)	0.539

Data were expressed as *n* (%), mean (±SD), and median (interquartile range).

The *p*-values compared between the training and the validation cohort.

### 3.2 Development of a nomogram for predicting early-onset PE

**Figure 2:**
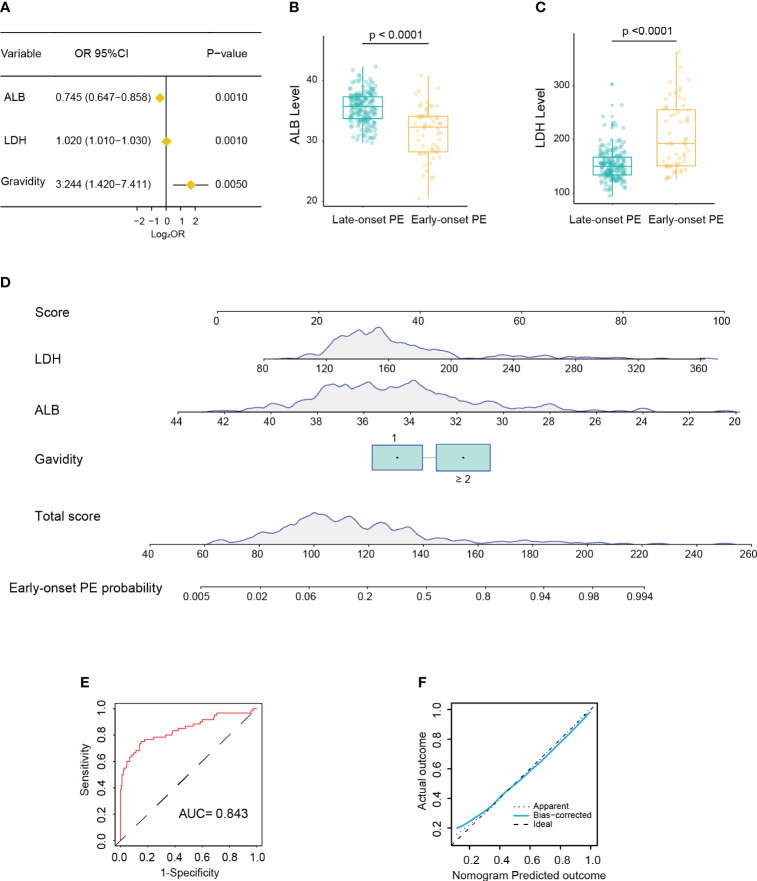
Development of a nomogram for predicting early-onset pre-eclampsia (PE). **(A)** Forest plot showing multiple gravidity experience, lower albumin (ALB), and higher lactate dehydrogenase (LDH) as significantly associated with early-onset PE in multivariable Cox regression analysis. **(B, C)** Box plot showing the distribution of the levels of ALB **(B)** and LDH **(C)** between the early-onset and late-onset PE groups (Wilcoxon test). **(D)** Nomogram for predicting early-onset PE probability in the training cohort. **(E)** Receiver operating characteristic (ROC) curve of the nomogram predicting early-onset PE probability in the training cohort. **(F)** Calibration curve of the nomogram predicting early-onset PE probability in the training cohort.

In the training cohort, 60 patients (23.7%) were diagnosed as early-onset PE. Univariate logistic analysis showed that patients with early-onset PE had multiple gravidity experience (*p* < 0.001); abortion history (*p* < 0.001); gemellary pregnancy (*p* = 0.021); elevated levels of MPV (*p* = 0.031), TT (*p* < 0.001), AST (*p* < 0.001), ALP (*p* < 0.001), LDH (*p* < 0.001), SAA (*p* = 0.006), and TBA (*p* < 0.001); and reduced levels of ALB (*p* < 0.001) (**Supplementary Table S1**). The above variables were incorporated into the multivariate logistic model and revealed that multiple gravidity experience (OR = 3.244, 95%CI = 1.420–7.411, *p* = 0.005), low ALB levels (32.3 *vs*. 35.7; OR = 0.745, 95%CI = 0.647–0.858, *p* < 0.001), and high LDH levels (192 *vs*. 150; OR = 1.020, 95%CI = 1.010–1.030, *p* < 0.001) were significantly associated with early-onset PE (**Figures 2A–C**). Based on these three variables, a nomogram was constructed to predict the probability of early-onset PE for each individual patient (**Figure 2D**). The C-index and AUC of the nomogram were both 0.843 (95%CI = 0.776–0.910), indicating good predictive performance. In addition, the calibration curve demonstrated good consistency between the probabilities predicted by the nomogram and the actual results (**Figures 2E, F**), and DCA showed that the nomogram offered a net benefit over the “treat-all” or “treat-none” strategy (**Supplementary Figure S1A**).

### 3.3 Development of a nomogram for predicting severe PE

**Figure 3:**
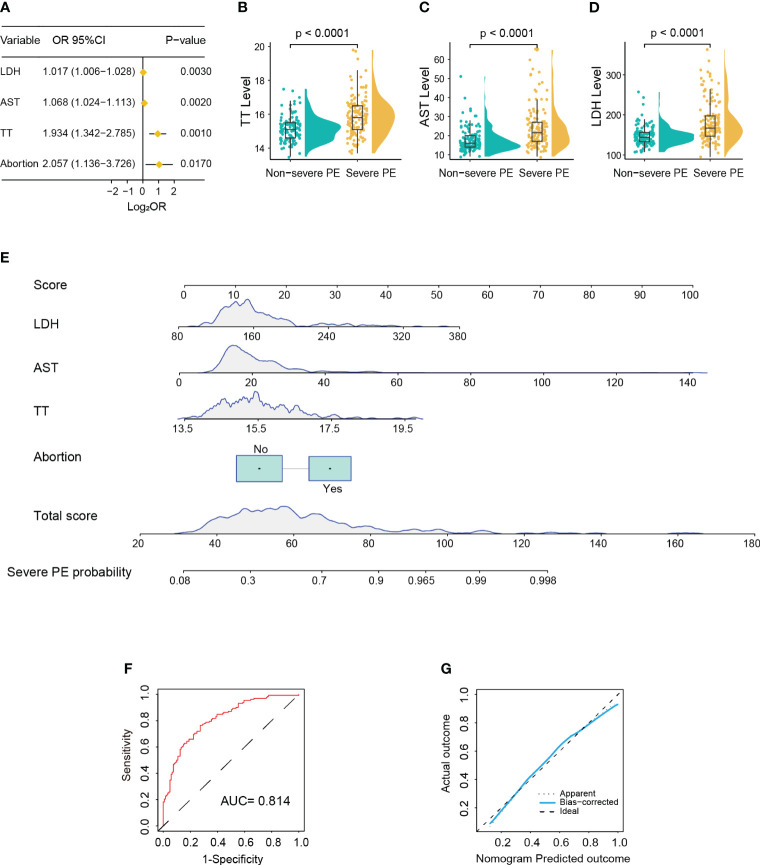
Development of a nomogram for predicting severe pre-eclampsia (PE). **(A)** Forest plot showing abortion history, prolonged thrombin time (TT), and higher aspartate aminotransferase (AST) and lactate dehydrogenase (LDH) as significantly associated with severe PE in multivariable Cox regression analysis. **(B–D)** Violin box plots showing the distribution of the levels of TT **(B)**, AST **(C)**, and LDH **(D)** between the severe and non-severe PE groups (Wilcoxon test). **(E)** Nomogram for predicting severe PE probability in the training cohort. **(F)** Receiver operating characteristic (ROC) curve of the nomogram predicting severe PE probability in the training cohort. **(G)** Calibration curve of the nomogram predicting severe PE probability in the training cohort.

In the training cohort, 132 patients (52.2%) were diagnosed as severe PE. Univariate logistic analysis showed that patients with early-onset PE had multiple gravidity experience (*p* < 0.001); abortion history (*p* < 0.001); elevated levels of Hb (*p* = 0.023), MPV (*p* = 0.001), TT (*p* = 0.001), AST (*p* < 0.001), ALP (*p* < 0.001), LDH (*p* = 0.003), and TBA (*p* = 0.048); and reduced levels of RDW (*p* = 0.030), PT (*p* = 0.025), and ALB (*p* < 0.001) (**Supplementary Table S2**). The above variables were incorporated into the multivariate logistic model and revealed that abortion history (OR = 2.057, 95%CI = 1.136–3.726, *p* = 0.017) and elevated levels of TT (15.9 *vs*. 15.1; OR = 1.934, 95%CI = 1.342–2.785, *p* < 0.001), AST (21.5 *vs*. 14.0; OR = 1.068, 95%CI = 1.024–1.113, *p* = 0.002), and LDH (167 *vs*. 144; OR = 1.017, 95%CI = 1.006–1.028, *p* = 0.003) were significantly associated with severe PE (**Figures 3A–D**). Based on these four variables, a nomogram was constructed to predict the probability of severe PE for each individual patient (**Figure 3E**). The C-index and AUC of the nomogram were both 0.814 (95%CI = 0.762–0.866), indicating good predictive performance. In addition, the calibration curve demonstrated good consistency between the probabilities predicted by the nomogram and the actual results (**Figures 3F, G**), and DCA showed that the nomogram offered a net benefit over the “treat-all” or “treat-none” strategy (**Supplementary Figure S1B**).

### 3.4 Development of a nomogram for predicting gestation duration

**Figure 4:**
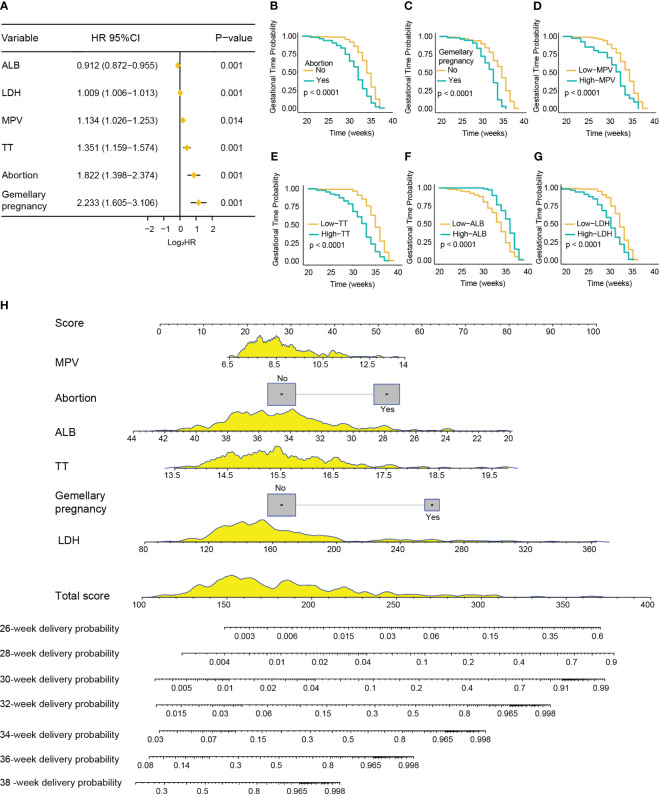
Development of a nomogram for predicting the gestation duration of patients with pre-eclampsia (PE). **(A)** Forest plot showing abortion history, gemellary pregnancy, prolonged thrombin time (TT), higher mean platelet volume (MPV) and lactate dehydrogenase (LDH), and lower albumin (ALB) as significantly associated with severe PE in multivariable Cox regression analysis. **(B–G)** Kaplan–Meier curves of gestation duration according to abortion history **(B)**, gemellary pregnancy **(C)**, MPV **(D)**, TT **(E)**, ALB **(F)**, and LDH **(G)** (log-rank test). **(H)** Nomogram for predicting the delivery probability of patients with PE in the training cohort.

**Figure 5:**
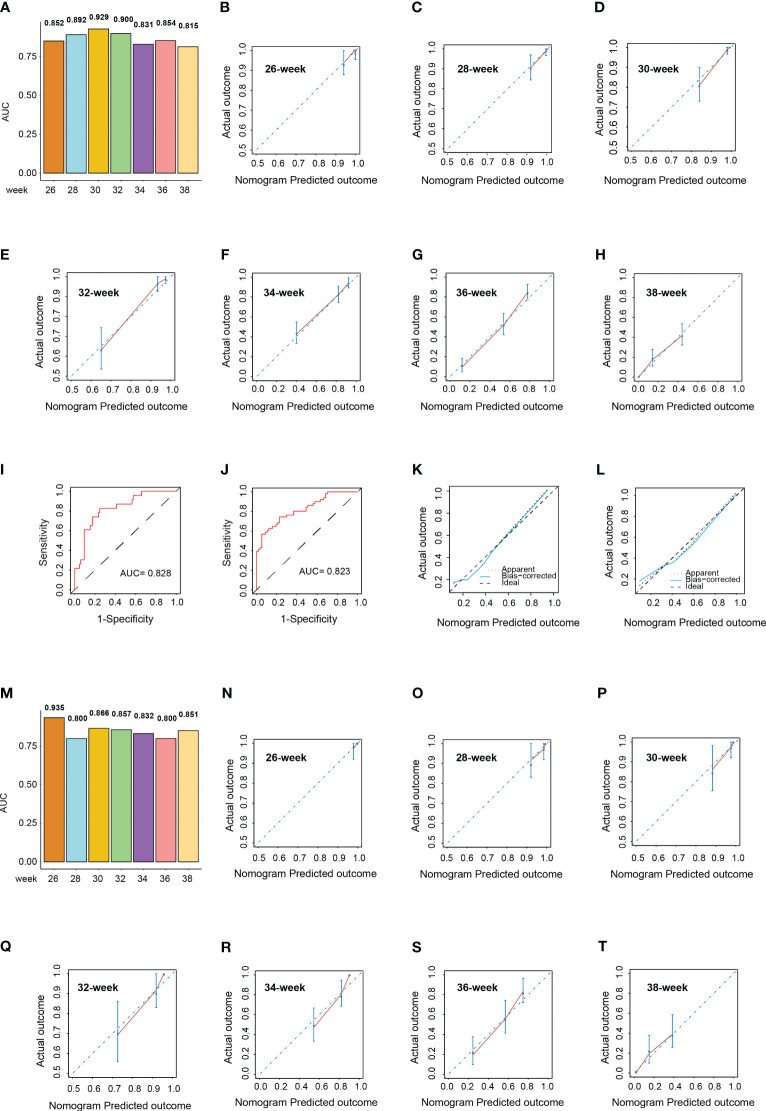
Validation of the nomograms in the validation cohort. **(A)** Area under the curve (AUC) of the nomogram predicting the delivery probability of patients with pre-eclampsia (PE) at 26, 28, 30, 32, 34, 36, and 38 weeks in the training cohort. **(B–H)** Calibration curves of the nomogram predicting the delivery probability of patients with PE at 26 **(B)**, 28 **(C)**, 30 **(D)**, 32 **(E)**, 34 **(F)**, 36 **(G)**, and 38 weeks **(H)** in the training cohort. **(I, J)** Receiver operating characteristic (ROC) curves of the nomograms predicting early-onset **(I)** and severe **(J)** PE probability in the validation cohort. **(K, L)** Calibration curves of the nomograms predicting early-onset **(K)** and severe **(L)** PE probability in the validation cohort. **(M)** AUCs of the nomogram predicting the delivery probability of patients with PE at 26, 28, 30, 32, 34, 36, and 38 weeks in the validation cohort. **(N–T)** Calibration curves of the nomogram predicting the delivery probability of patients with PE at 26 **(N)**, 28 **(O)**, 30 **(P)**, 32 **(Q)**, 34 **(R)**, 36 **(S)**, and 38 weeks **(T)** in the validation cohort.

In the training cohort, the median gestation duration was 36.0 weeks (range, 34.0–38.0 weeks). The results of the univariate Cox analysis showed that higher age (*p* < 0.001); multiple gravidity experience (*p* < 0.001); abortion history (*p* < 0.001); gemellary pregnancy (*p* < 0.001); multipara (*p* = 0.001); elevated levels of MPV (*p* < 0.001), TT (*p* < 0.001), AST (*p* = 0.002), ALP (*p* < 0.001), LDH (*p* < 0.001), SAA (*p* = 0.015), TBA (*p* = 0.007), and CRP (*p* = 0.004); and reduced levels of ALB (*p* < 0.001) were associated with shorter gestation duration (**Supplementary Table S3**). The above variables were incorporated into the multivariate Cox model and revealed that abortion history (HR = 1.822, 95%CI = 1.398–2.374, *p* < 0.001); gemellary pregnancy (HR = 2.233, 95%CI = 1.605–3.106, *p* < 0.001); elevated levels of MPV (HR = 1.134, 95%CI = 1.026–1.253, *p* = 0.014), TT (HR = 1.351, 95%CI = 1.159–1.574, *p* < 0.001), and LDH (HR = 1.009, 95%CI = 1.006–1.013, *p* < 0.001); and reduced levels of ALB (HR = 0.912, 95%CI = 0.872–0.955, *p* < 0.001) were significantly associated with shorter gestation duration (**Figures 4A–G**). Based on these six variables, a nomogram was constructed to predict the probability of delivery at 26, 28, 30, 32, 34, 36, and 38 gestational weeks for each individual PE patient (**Figure 4H**). The C-index of the nomogram was 0.772 (95%CI = 0.740–0.804). The AUC of each time point of nomogram prediction demonstrated good performance and discrimination (26 weeks: 0.852, 95%CI = 0.757–0.914; 28 weeks: 0.892, 95%CI = 0.730–0.942; 30 weeks: 0.929, 95%CI = 0.792–0.981; 32 weeks: 0.900, 95%CI = 0.881–0.955; 34 weeks: 0.831, 95%CI = 0.721–0.916; 36 weeks: 0.854, 95%CI = 0.718–0.935; 38 weeks: 0.815, 95%CI = 0.701–0.924) (**Figure 5A**). The time-dependent ROC curve is shown in **Supplementary Figure S2A**. Furthermore, the calibration curve demonstrated good consistency between the nomogram’s predicted probabilities and the actual results (**Figures 5B–H**), and DCA showed that the nomogram offered a net benefit over the “treat-all” or “treat-none” strategy at each time point (**Supplementary Figures S1C–I**).

### 3.5 Validation of the nomograms

In the validation cohort, the median age was 32.0 years (range, 28.0–37.0 years). There were 58 patients (53.7%) who had multiple gravidity experience and 72 patients (66.7%) who were primiparas. A total of 85 patients (78.7%) had a single pregnancy, 48 patients (44.4%) had a history of abortion, and 90 patients (83.3%) had irregular menstruation.

In the validation cohort, there were 23 patients (21.3%) with early-onset PE and 51 patients (47.2%) with severe PE. The AUCs of the nomogram predicting early-onset PE and severe PE were 0.828 (95%CI = 0.736–0.919) and 0.823 (95%CI = 0.746–0.901), respectively (**Figures 5I, J**). The median gestation duration in the validation cohort was 37.0 weeks (range, 35.0–38.0 weeks). The C-index of the nomogram was 0.741 (95%CI = 0.689–0.793). The AUC of each time point of nomogram prediction demonstrated good performance and discrimination (26 weeks: 0.935, 95%CI = 0.872–0.977; 28 weeks: 0.800, 95%CI = 0.734–0.901; 30 weeks: 0.866, 95%CI = 0.716–0.923; 32 weeks: 0.857, 95%CI = 0.696–0.953; 34 weeks: 0.832, 95%CI = 0.724–0.938; 36 weeks: 0.800, 95%CI = 0.749–0.910; 38 weeks: 0.851, 95%CI = 0.772–0.924) (**Figure 5M**). The time-dependent ROC curve in the validation cohort is shown in **Supplementary Figure S2B**. The calibration curves for each nomogram also demonstrated good consistency between the predicted probabilities and the actual results (**Figures 5K, L, N–T**), and DCA showed that the nomogram offered a net benefit over the “treat-all” or “treat-none” strategy at each time point (**Supplementary Figures S1L–R**).

## 4 Discussion

In this study, based on the clinical characteristics and peripheral blood laboratory indicators, we identified a series of risk factors associated with early-onset PE, severe PE, and shorter gestation duration of patients with PE. In addition, three nomograms were developed to predict the probability of early-onset PE, severe PE, and delivery at different gestational weeks for each individual patient with PE, which could help clinicians manage patients with PE in the early stage of the disease and improve the clinical outcomes for pregnant women and infants.

The etiologies and pathogenesis of PE are complex and multisystemic, which involve placental dysfunction, immune system dysfunction, maternal metabolic disorder, and dysregulated endothelial function due to the release of a series of circulating factors including angiogenic proteins, pro-inflammatory cytokines, and small extracellular vesicles (25–30). Several risk factors of obstetric history have been identified as associated with PE from clinical guidelines, including previous PE, history of parity, gravidity, abortion, and multiple pregnancies, which were considered to lead to a weakened maternal immune tolerance to the placenta, thus increasing the risk of PE (3, 7, 8). A previous study found that multiple fetal pregnancies were associated with a significantly higher rate of PE than singleton pregnancies, with the rate increasing with the number of fetuses present (31). In this study, multiple gravidities, previous abortion history, and multiple pregnancies were also found to be independent risk factors for inferior clinical outcomes of patients with PE, which is consistent with previous results.

Accumulating evidence suggested that impaired maternal metabolic function is associated with PE, which leads to inadequate adaptation to the demands of pregnancy. An altered metabolic function has been proposed to contribute to PE by causing reduced spiral artery remodeling and altered placental metabolic function (28). A previous study evaluated the role of LDH isozymes in the placenta between patients with PE and those with normal pregnancy and found that, compared to placentas from normal pregnancy, the mRNA and activity of LDH-A were increased in placentas from patients with PE, probably as a result of hypoxia (32). In this study, we found that a higher serum LDH level was related to early-onset PE, severe PE, and shorter gestation duration of patients with PE, indicating that LDH could serve as a marker for PE.

In addition, there was an increase of transaminases and hypoalbuminemia in PE patients with poor clinical outcomes, suggesting that an impaired liver function was associated with severe PE and shorter gestation duration of patients with PE. Similarly, several researchers also observed impaired liver function in patients with PE, including elevated AST and ALT and reduced ALB (33, 34). This elevation may be due to the systemic inflammatory response caused by placental ischemia, which then resulted in vasoconstriction and endothelial dysfunction and eventual liver dysfunctions.

Platelet activation occurred in the early stage of PE, which may be associated with platelet aggregation and depletion due to injury to the vascular endothelium in patients with PE (35). In the present study, we found that a higher MPV was related to the shorter gestation duration of patients with PE. High levels of MPV represented a high platelet consumption status, and this aggregation and depletion would result in increased blood viscosity and the potential for microthrombosis, which could also lead to placental ischemia and hypoxia and further affect both maternal organ function and fetus growth. Moreover, we found that a prolonged TT was associated with the shorter gestation duration of patients with PE, suggesting that the coagulation function disorder could affect the severity of PE. Thrombin time refers to the time it takes for thrombin to convert fibrinogen into fibrin; the prolonged TT reflected the insufficiency of plasma fibrinogen or abnormal structure, or excessive anticoagulant substances in the body. A previous study identified that the levels of fibrinogen were significantly lower in placentas from women with early-onset PE compared with control placentas, indicating that a low fibrinogen level might be involved in the coagulation disorder in PE (36).

Although numerous studies have explored a series of risk factors related to PE, none have constructed nomograms to accurately predict the probabilities of early-onset PE, severe PE, and the gestation duration of patients with PE. Here, based on significant clinical features and peripheral blood laboratory indicators, we constructed three nomograms to predict the probability of early-onset PE, severe PE, and delivery at 26, 28, 30, 32, 34, 36, and 38 weeks for pregnant women with PE. The AUC of each nomogram presented good discriminative ability, and each nomogram was validated in the validation cohort. Although there were several flaws in the calibration curves at 26–30 weeks, we believe that the main reason was the improvements in medical condition and early intervention, and thus the delivery events were relatively low. However, the results of the AUC and C-index, as well as the DCA, demonstrated that our model still had reliability for prediction. Compared with other reported biomarkers or risk scores, the model developed in this study could quantify each predictive variable and provide a specific probability of the occurrence of severe and early-onset PE and the delivery time for each individual pregnant woman. Furthermore, this model could also help clinicians assess the clinical outcomes early and design an appropriate strategy for each patient. To our knowledge, this is the first study to construct three different nomograms to predict early-onset PE, severe PE, and the gestation duration of patients with PE based on clinical characteristics and laboratory parameters.

This study has some limitations. Firstly, it is a retrospective study with a relatively small sample size. Therefore, expanding the sample size and assessing the predictive performance of the nomograms in a larger prospective study are needed. Secondly, this study did not examine the serum sFlt-1, PlGF, PP13, PAPP-A, and VCAM-1, which are considered as important markers for PE.

## 5 Conclusion

Based on the clinical features and peripheral blood laboratory indicators, we identified significant factors and developed models to predict early-onset PE, severe PE, and the gestation duration of pregnant women with PE, which could help clinicians assess the clinical outcomes early and design an appropriate strategy for each patient.

## Data availability statement

The original contributions presented in the study are included in the article/**Supplementary Material**. Further inquiries can be directed to the corresponding authors.

## Ethics statement

The studies involving humans were approved by the Ethics Committee of Sir Run Run Shaw Hospital (The Approval No. 2023-0248). The studies were conducted in accordance with the local legislation and institutional requirements. Written informed consent for participation in this study was provided by the participants’ legal guardians/next of kin.

## Author contributions

YX: Data curation, Formal analysis, Investigation, Methodology, Software, Visualization, Writing – original draft. YW: Data curation, Formal analysis, Methodology, Writing – original draft. SY: Formal analysis, Methodology, Writing – original draft. JHy: Data curation, Software, Writing – original draft. LZ: Data curation, Formal analysis, Software, Writing – original draft. JX: Data curation, Formal analysis, Software, Writing – original draft. YZ: Data curation, Software, Writing – original draft. JHa: Data curation, Formal analysis, Software, Writing – original draft. YR: Conceptualization, Supervision, Writing – review & editing. SW: Conceptualization, Supervision, Writing – review & editing.

## Supplementary material

Supplementary Figure 1 Decision curve analysis of three nomograms. **(A, J)** Decision curve analysis of nomogram for predicting early-onset PE in training cohort **(A)** and validation cohort **(J)**. **(B, K)** Decision curve analysis of nomogram for predicting severe PE in training cohort **(B)** and validation cohort **(K)**. **(C–I, L-R)** Decision curve analysis of nomogram for predicting delivery probability of PE patients at 26-, 28-, 30-, 32-, 34-, 36-, and 38-week in training cohort **(C–I)** and validation cohort **(L–R)**. The y-axis indicates the net benefit, which is the sum of the benefits (true positives) minus harm (false positives). The x-axis indicates the threshold probability. The red line represents the nomogram net benefit. The green and black lines represent the hypotheses that all or no patients occurred end point event, respectively.

Supplementary Figure 2 The time-dependent ROC curve of nomogram for predicting delivery probability of PE patients. **(A, B)** The time-dependent ROC curve of nomogram for predicting delivery probability of PE patients at 26-38 week in training cohort **(A)** and validation cohort **(B)**.
