# Supplementary figures and images for: Development and validation of nomograms to predict clinical outcomes of preeclampsia
Source: Front Endocrinol (Lausanne). 2024 Mar 14;15:1292458. doi: 10.3389/fendo.2024.1292458 (PMC10972945; doi:10.3389/fendo.2024.1292458)

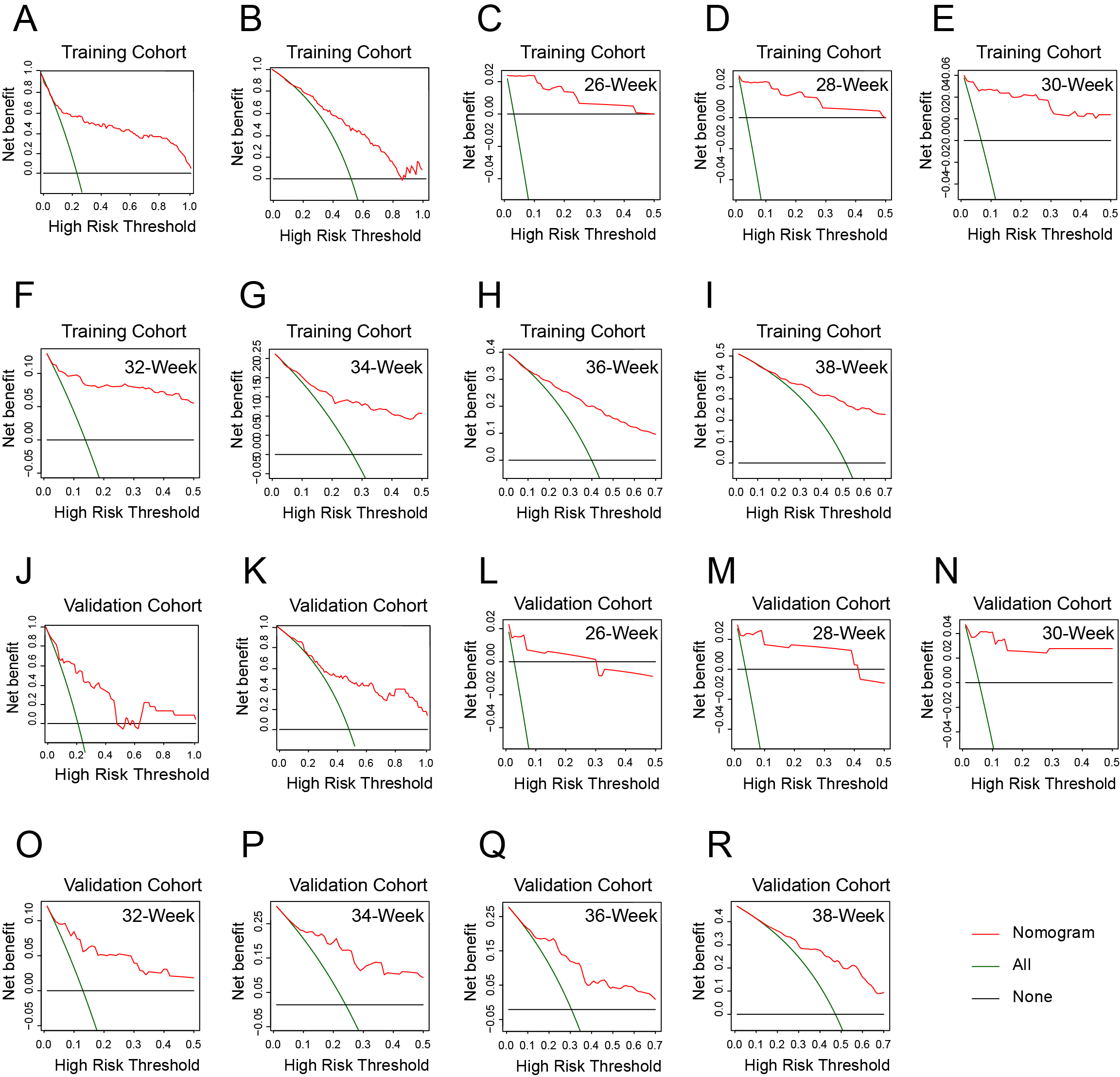

Supplement: Supplementary Figure 1 — Decision curve analysis of three nomograms. (A, J) Decision curve analysis of nomogram for predicting early-onset PE in training cohort (A) and validation cohort (J). (B, K) Decision curve analysis of nomogram for predicting severe PE in training cohort (B) and validation cohort (K). (C–I, L-R) Decision curve analysis of nomogram for predicting delivery probability of PE patients at 26-, 28-, 30-, 32-, 34-, 36-, and 38-week in training cohort (C–I) and validation cohort (L–R). The y-axis indicates the net benefit, which is the sum of the benefits (true positives) minus harm (false positives). The x-axis indicates the threshold probability. The red line represents the nomogram net benefit. The green and black lines represent the hypotheses that all or no patients occurred end point event, respectively. [file Image_1.tif]

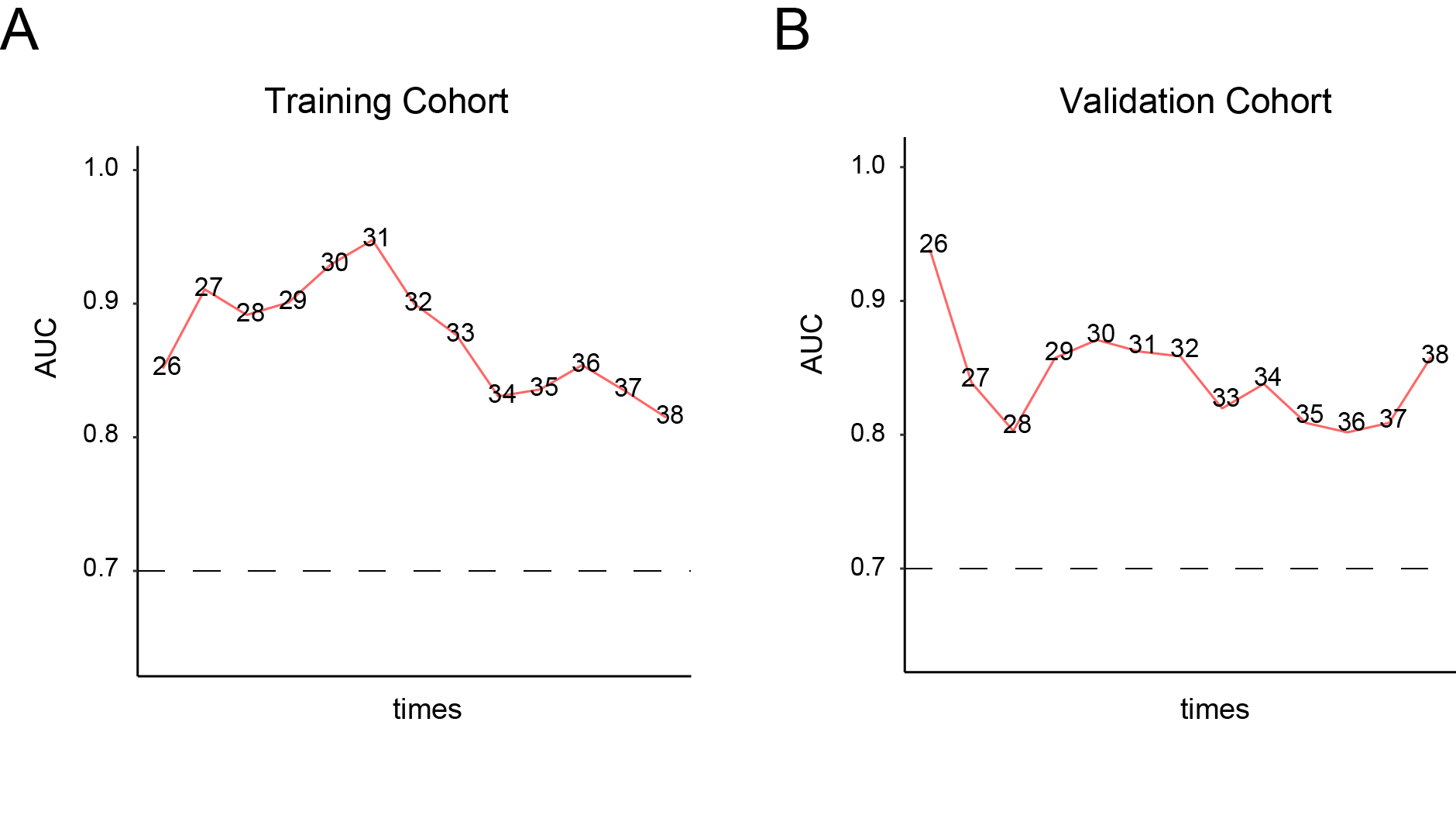

Supplement: Supplementary Figure 2 — The time-dependent ROC curve of nomogram for predicting delivery probability of PE patients. (A, B) The time-dependent ROC curve of nomogram for predicting delivery probability of PE patients at 26-38 week in training cohort (A) and validation cohort (B). [file Image_2.tif]
