# Supplementary material for: Development and validation of nomograms to predict clinical outcomes of preeclampsia
Source: Front Endocrinol (Lausanne). 2024 Mar 14;15:1292458. doi: 10.3389/fendo.2024.1292458 (PMC10972945; doi:10.3389/fendo.2024.1292458)
Supplement: Supplementary file 3 [file Table_1.docx]

**Table S1. Univariable Logistic regression analysis for early-onset PE.**

| **Variable** | **Early-onset PE (n=60)** | **Late-onset PE (n=193)** | **P-value** |
| --- | --- | --- | --- |
| **Clinical characteristics** |  |  |  |
| Age, years (IQR) | 34.0 (30.8, 36.0) | 32.0 (28.0, 35.0) | 0.054 |
| Gravidity |  |  | <0.001 |
| 1 | 10 (16.7%) | 108 (56.0%) |  |
| ≥2 | 50 (83.3%) | 85 (44.0%) |  |
| Parity |  |  | 0.167 |
| Primipara | 40 (66.7%) | 148 (76.7%) |  |
| Multipara | 20 (33.3%) | 45 (23.3%) |  |
| Abortion |  |  | <0.001 |
| No | 17 (28.3%) | 120 (62.2%) |  |
| Yes | 43 (71.7%) | 73 (37.8%) |  |
| Gemellary pregnancy |  |  | 0.021 |
| No | 40 (66.7%) | 158 (81.9%) |  |
| Yes | 20 (33.3%) | 35 (18.1%) |  |
| Menstrual regularity |  |  | 0.17 |
| No | 4 (6.67%) | 28 (14.5%) |  |
| Yes | 56 (93.3%) | 165 (85.5%) |  |
| **Laboratory parameters** |  |  |  |
| WBC, ×10⁹/L | 9.92 (2.49) | 10.0 (2.22) | 0.808 |
| RBC, ×10^12^/L | 3.84 (0.45) | 3.84 (0.39) | 0.978 |
| Hb, g/L | 111 (12.5, 123) | 105 (11.9, 121) | 0.224 |
| Hematocrit, % | 35.2 (32.7, 37.9) | 35.3 (33.2, 36.8) | 0.481 |
| MCV, fL | 92.6 (90.0, 94.9) | 91.4 (88.2, 94.5) | 0.098 |
| PC, ×10⁹/L | 212 (183, 246) | 212 (175, 256) | 0.606 |
| ANC, ×10⁹/L | 7.43 (6.19, 8.77) | 7.30 (6.21, 9.00) | 0.908 |
| ALC, ×10⁹/L | 1.64 (1.40, 1.97) | 1.70 (1.43, 2.00) | 0.485 |
| AMC, ×10⁹/L | 0.50 (0.41, 0.67) | 0.51 (0.40, 0.62) | 0.811 |
| AEC, ×10⁹/L | 0.07 (0.02, 0.10) | 0.09 (0.04, 0.10) | 0.125 |
| ABC, ×10⁹/L | 0.01 (0.00, 0.03) | 0.02 (0.00, 0.03) | 0.566 |
| RDW, % | 13.4 (13.0, 13.8) | 13.5 (13.0, 14.0) | 0.149 |
| PDW, % | 16.8 (16.5, 17.3) | 16.8 (16.2, 17.3) | 0.628 |
| MPV, fL | 8.80 (7.88, 10.1) | 8.40 (7.80, 9.20) | 0.031 |
| Thrombocytocrit, % | 0.18 (0.17, 0.21) | 0.18 (0.16, 0.21) | 0.241 |
| PT, s | 12.3 (12.0, 12.7) | 12.5 (12.1, 12.8) | 0.286 |
| INR | 0.92 (0.90, 0.96) | 0.93 (0.91, 0.96) | 0.087 |
| APTT, s | 31.9 (30.0, 32.9) | 31.5 (29.8, 33.1) | 0.809 |
| TT, s | 16.1 (15.4, 16.6) | 15.2 (14.6, 15.8) | <0.001 |
| Fibrinogen, g/L | 4.52 (0.90) | 4.74 (0.71) | 0.087 |
| ALT, U/L | 14.0 (10.8, 22.0) | 13.0 (10.0, 19.0) | 0.392 |
| AST, U/L | 24.0 (17.8, 30.5) | 17.0 (14.0, 22.0) | <0.001 |
| ALP, U/L | 87.5 (68.8, 107) | 71.0 (58.0, 85.0) | <0.001 |
| Albumin, g/L | 32.3 (28.2, 34.1) | 35.7 (33.7, 37.3) | <0.001 |
| LDH, U/L | 192 (151, 256) | 150 (134, 167) | <0.001 |
| SAA, mg/L | 5.80 (4.20, 9.82) | 4.60 (3.00, 7.30) | 0.006 |
| TBA, μmol/L | 3.17 (2.02, 4.68) | 2.24 (1.46, 3.25) | <0.001 |
| CRP, mg/L | 2.70 (1.75, 5.90) | 3.10 (1.80, 5.20) | 0.795 |
