# Supplementary material for: Development and validation of nomograms to predict clinical outcomes of preeclampsia
Source: Front Endocrinol (Lausanne). 2024 Mar 14;15:1292458. doi: 10.3389/fendo.2024.1292458 (PMC10972945; doi:10.3389/fendo.2024.1292458)
Supplement: Supplementary file 4 [file Table_2.docx]

**Table S2. Univariable Logistic regression analysis for severe PE.**

| **Variable** | **Non-severe PE (n=121)** | **Severe PE (n=132)** | **P-value** |
| --- | --- | --- | --- |
| **Clinical characteristics** |  |  |  |
| Age, years (IQR) | 32.0 (28.0, 35.0) | 33.0 (29.0, 36.0) | 0.061 |
| Gravidity |  |  | <0.001 |
| 1 | 71 (58.7%) | 47 (35.6%) |  |
| ≥2 | 50 (41.3%) | 85 (64.4%) |  |
| Parity |  |  | 0.648 |
| Primipara | 92 (76.0%) | 96 (72.7%) |  |
| Multipara | 29 (24.0%) | 36 (27.3%) |  |
| Abortion |  |  | <0.001 |
| No | 84 (69.4%) | 53 (40.2%) |  |
| Yes | 37 (30.6%) | 79 (59.8%) |  |
| Gemellary pregnancy |  |  | 0.077 |
| No | 101 (83.5%) | 97 (73.5%) |  |
| Yes | 20 (16.5%) | 35 (26.5%) |  |
| Menstrual regularity |  |  | 0.651 |
| No | 17 (14.0%) | 15 (11.4%) |  |
| Yes | 104 (86.0%) | 117 (88.6%) |  |
| **Laboratory parameters** |  |  |  |
| WBC, ×10⁹/L | 10.2 (2.23) | 9.76 (2.32) | 0.104 |
| RBC, ×10^12^/L | 3.82 (0.37) | 3.86 (0.44) | 0.415 |
| Hb, g/L | 101 (11.7, 120) | 112 (12.4, 123) | 0.023 |
| Hematocrit, % | 35.1 (32.7, 36.6) | 35.5 (33.7, 37.3) | 0.133 |
| MCV, fL | 91.4 (87.7, 93.5) | 92.0 (89.2, 95.0) | 0.087 |
| PC, ×10⁹/L | 213 (188, 255) | 210 (166, 249) | 0.124 |
| ANC, ×10⁹/L | 7.50 (6.40, 9.10) | 7.12 (5.96, 8.84) | 0.091 |
| ALC, ×10⁹/L | 1.71 (1.50, 2.00) | 1.64 (1.38, 2.00) | 0.165 |
| AMC, ×10⁹/L | 0.52 (0.41, 0.62) | 0.50 (0.40, 0.65) | 0.677 |
| AEC, ×10⁹/L | 0.09 (0.04, 0.10) | 0.08 (0.04, 0.10) | 0.968 |
| ABC, ×10⁹/L | 0.01 (0.00, 0.03) | 0.02 (0.00, 0.03) | 0.084 |
| RDW, % | 13.6 (13.2, 14.1) | 13.4 (12.9, 13.9) | 0.030 |
| PDW, % | 16.7 (16.3, 17.3) | 16.9 (16.4, 17.4) | 0.115 |
| MPV, fL | 8.30 (7.50, 8.90) | 8.90 (8.60, 10.1) | 0.001 |
| Thrombocytocrit, % | 0.18 (0.16, 0.22) | 0.18 (0.16, 0.21) | 0.300 |
| PT, s | 12.5 (12.2, 13.0) | 12.3 (11.6, 12.6) | 0.025 |
| INR | 0.93 (0.91, 0.97) | 0.93 (0.90, 0.96) | 0.165 |
| APTT, s | 31.3 (29.6, 33.2) | 31.7 (30.2, 32.9) | 0.457 |
| TT, s | 15.1 (14.7, 15.7) | 15.9 (15.4, 16.6) | 0.001 |
| Fibrinogen, g/L | 4.75 (0.75) | 4.63 (0.77) | 0.222 |
| ALT, U/L | 13.0 (10.0, 18.0) | 13.0 (10.0, 23.0) | 0.403 |
| AST, U/L | 14.0 (16.0, 20.0) | 21.5 (17.0, 27.0) | <0.001 |
| ALP, U/L | 69.0 (56.0, 83.0) | 77.5 (64.8, 99.0) | <0.001 |
| Albumin, g/L | 34.7 (2.06) | 32.5 (1.90) | <0.001 |
| LDH, U/L | 144 (132, 156) | 167 (146, 198) | 0.003 |
| SAA, mg/L | 5.00 (3.40, 7.50) | 4.70 (3.18, 8.10) | 0.836 |
| TBA, μmol/L | 2.52 (1.44, 3.25) | 2.50 (1.70, 3.85) | 0.048 |
| CRP, mg/L | 3.00 (1.70, 4.90) | 3.00 (1.80, 5.73) | 0.865 |
