# Supplementary material for: Development and validation of nomograms to predict clinical outcomes of preeclampsia
Source: Front Endocrinol (Lausanne). 2024 Mar 14;15:1292458. doi: 10.3389/fendo.2024.1292458 (PMC10972945; doi:10.3389/fendo.2024.1292458)
Supplement: Supplementary file 5 [file Table_3.docx]

**Table S3. Univariable Logistic regression analysis for gestation duration of PE patients.**

| **Variable** | **HR (95% CI)** | **P-value** |
| --- | --- | --- |
| **Clinical characteristics** |  |  |
| Age, years | 1.053 (1.027-1.080) | <0.001 |
| Gravidity |  |  |
| 1 | 1 [Reference] |  |
| ≥2 | 2.049 (1.591-2.638) | <0.001 |
| Parity |  |  |
| Primipara | 1 [Reference] |  |
| Multipara | 1.654 (1.237-2.211) | 0.001 |
| Abortion |  |  |
| No | 1 [Reference] |  |
| Yes | 2.161 (1.677-2.785) | <0.001 |
| Gemellary pregnancy |  |  |
| No | 1 [Reference] |  |
| Yes | 3.261 (2.343-4.537) | <0.001 |
| Menstrual regularity |  |  |
| No | 1 [Reference] |  |
| Yes | 0.952 (0.656-1.381) | 0.795 |
| **Laboratory parameters** |  |  |
| WBC, ×10⁹/L | 1.004 (0.950-1.060) | 0.897 |
| RBC, ×10^12^/L | 1.126 (0.817-1.550) | 0.469 |
| Hb, g/L | 1.000 (0.998-1.003) | 0.699 |
| Hematocrit, % | 1.031 (0.984-1.081) | 0.195 |
| MCV, fL | 1.004 (0.981-1.029) | 0.722 |
| PC, ×10⁹/L | 0.999 (0.997-1.001) | 0.374 |
| ANC, ×10⁹/L | 1.009 (0.947-1.075) | 0.787 |
| ALC, ×10⁹/L | 0.886 (0.665-1.180) | 0.408 |
| AMC, ×10⁹/L | 1.304 (0.596-2.851) | 0.507 |
| AEC, ×10⁹/L | 1.063 (0.296-3.813) | 0.925 |
| ABC, ×10⁹/L | 0.219 (0.002-30.202) | 0.546 |
| RDW, % | 1.045 (0.956-1.143) | 0.332 |
| PDW, % | 1.025 (0.955-1.099) | 0.498 |
| MPV, fL | 1.202 (1.089-1.327) | <0.001 |
| Thrombocytocrit, % | 2.959 (0.144-60.834) | 0.482 |
| PT, s | 0.865 (0.679-1.102) | 0.241 |
| INR | 0.157 (0.011-2.221) | 0.171 |
| APTT, s | 0.987 (0.934-1.043) | 0.642 |
| TT, s | 1.811 (1.560-2.102) | <0.001 |
| Fibrinogen, g/L | 1.021 (0.859-1.214) | 0.811 |
| ALT, U/L | 1.001 (0.996-1.007) | 0.633 |
| AST, U/L | 1.010 (1.004-1.017) | 0.002 |
| ALP, U/L | 1.012 (1.009-1.016) | <0.001 |
| Albumin, g/L | 0.848 (0.811-0.887) | <0.001 |
| LDH, U/L | 1.014 (1.011-1.017) | <0.001 |
| SAA, mg/L | 1.006 (1.001-1.011) | 0.015 |
| TBA, μmol/L | 1.012 (1.003-1.020) | 0.007 |
| CRP, mg/L | 1.034 (1.010-1.058) | 0.004 |
